# Characterizing Family Abuse in Suicidal Ideation Posts on Reddit: Large Language Model–Assisted Content Analysis

**DOI:** 10.2196/90340

**Published:** 2026-09-14

**Authors:** Salim Sazzed

**Affiliations:** 1 Georgia Southern University Statesboro, GA United States

**Keywords:** suicidal ideation, family abuse, domestic violence, social media, Reddit, mental health, large language models

## Abstract

**Background:**

Suicidal ideation is a significant global public health concern, with family abuse recognized as a key risk factor. Although clinical studies have examined the link between family abuse and mental health, its representation in social media discussions—particularly in relation to suicidal ideation—remains largely unexplored.

**Objective:**

This study aimed to examine how family abuse manifests in suicidal ideation narratives on social media, focusing on the demographic characteristics of individuals reporting abuse, the types of abuse described, and relationships to perpetrators.

**Methods:**

We analyzed 27,434 posts from the Reddit *SuicideWatch* forum to identify posts in which suicidal ideation and family abuse co-occurred. Using a combination of manual review, large language model–assisted keyword expansion, natural language processing–based information extraction, and human validation, we examined self-disclosed age and gender among Reddit users who reported family abuse in suicidal ideation posts, along with abuse types and relationships to perpetrators.

**Results:**

Among posts with self-reported age information, individuals aged 18 to 24 years accounted for the largest proportion (144/313, 46.0%). Among posts with self-reported gender information, men represented 57.3% (86/150). Physical abuse was the most frequently reported form of family abuse (683/975, 70.1%; 95% CI 67.18%-72.93%), followed by emotional or psychological abuse (350/975, 35.9%; 95% CI 32.89%-38.91%) and sexual abuse (296/975, 30.4%; 95% CI 27.47%-33.25%). The parent category was the most frequently identified perpetrator category (435/975, 44.6%; 95% CI: 41.52%-47.75%).

**Conclusions:**

This study enhances our understanding of how family abuse appears in social media posts involving suicidal ideation. The findings demonstrate the potential of computational social media analysis to support descriptive, hypothesis-generating research and inform the design of online support resources for family abuse–related suicidal distress.

## Introduction

### Background

Suicidal ideation is a major public health concern and a leading cause of death, particularly among young individuals [1]. According to the World Health Organization, approximately 700,000 people die by suicide each year [2]. Suicidal ideation arises from a complex interplay of psychological, social, and environmental factors, with profound consequences for individuals, families, and communities [3,4]. Among these factors, family abuse is recognized as a significant contributor to suicidal ideation [5,6]. Family abuse—encompassing physical, emotional, and psychological harm—induces chronic stress and emotional instability, severely affecting mental health [7]. Survivors often report helplessness, low self-worth, and social isolation, all of which are strongly associated with suicidal ideation [8,9]. The relationship between family abuse and suicidal ideation is complex and multidimensional, influenced by intersecting factors such as the severity of abuse, the presence of protective social supports, and individual psychological resilience [10,11]. Understanding these intricate dynamics is essential for advancing our knowledge of suicide risk and developing nuanced interventions tailored to those affected by familial abuse. Existing clinical and survey-based studies demonstrate the profound impact of family abuse on mental health and its strong association with suicidal behaviors [12-14].

Individuals with a history of family abuse are at higher risk for psychological distress and suicidal ideation [8,15,16]. Joiner’s [3] interpersonal theory of suicide highlights perceived burdensomeness and thwarted belongingness as critical risk factors, both exacerbated by abusive experiences. Intimate partner violence, a key form of family abuse, has been linked to severe psychological consequences [5]. Furthermore, the adverse childhood experiences (ACEs) study, along with systematic reviews and meta-analyses, underscore the long-term effects of childhood maltreatment on suicide risk [8,17-19]. Research by Duprey et al [6] and Charak et al [7] further demonstrates the correlation between early abuse and increased risk of suicidal ideation and attempts across the lifespan. Studies have also examined vulnerabilities in specific populations: Kwok et al [9] identified adolescents as particularly susceptible to the effects of childhood maltreatment, while Kaplan et al [11] explored the experiences of adolescent suicide attempters with a history of physical abuse. Together, these studies emphasize the multifaceted nature of family abuse and the need for targeted, nuanced approaches to understanding its psychological and behavioral consequences. Unlike clinical assessments, social media mental health posts provide largely unfiltered, freeform accounts of distress and experiences of family abuse, offering insights that may not emerge in formal assessments. The growing body of research examining mental health through digital platforms has highlighted the unique potential of online spaces to reveal critical insights into individuals’ lived experiences [20,21]. Social media platforms, which are increasingly used as outlets for expressing emotional distress, offer an unprecedented opportunity to study the real-time manifestations of suicidal ideation and its associated triggers [22-25]. Through the analysis of social media health data, researchers can gain critical insights into the various factors that shape suicidal thoughts, ultimately contributing to the development of evidence-based strategies for prevention and intervention [26]. However, despite recent progress, gaps remain in understanding the multifaceted nature and risk factors of suicidal ideation, including the dynamics of family abuse within online communities and discourses.

To address this gap, this study explores the intersection of family abuse and suicidal ideation in online discourse by analyzing textual content from the Reddit (Reddit, Inc) forum *r/SuicideWatch*. We aim to uncover the multidimensional nature of family abuse among individuals reporting suicidal thoughts by addressing the following three research questions (RQs):

RQ1 addresses the demographic characteristics of individuals reporting family abuse. This research question examines the demographic distributions of individuals who report experiencing family abuse alongside suicidal ideation by extracting attributes such as gender and age. Understanding these characteristics is essential for identifying vulnerable groups and informing the development of tailored interventions.

RQ2 addresses the relationships to perpetrators. What are the relationships to perpetrators in cases of family abuse, including roles such as parent (eg, father or mother), sibling, or intimate partner (eg, boyfriend or spouse)? Examining these relationships is critical for identifying the dynamics that contribute to suicidal ideation and informing targeted intervention strategies.

RQ3 addresses the types of family abuse. What is the prevalence of different forms of family abuse (eg, physical and emotional), and what specific characteristics define each type?

Understanding these patterns clarifies the range of harm individuals experience and informs the development of more comprehensive and targeted interventions.

To explore these research questions, we analyze social media posts related to suicidal ideation written by individuals reporting experiences of family abuse. Using a dataset of approximately 27,434 posts collected from the Reddit forum *r/SuicideWatch*, a keyword-based search was applied to identify about 975 posts that explicitly describe experiences of family abuse. We then examine key demographic characteristics of individuals reporting family abuse; explore relationships to perpetrators; and identify prevalent forms of abuse, including physical, emotional, and sexual abuse, as well as neglect. Our findings reveal demographic distributions and the prevalence and forms of family abuse across different groups. These insights enhance our understanding of family abuse in relation to suicidal ideation and can inform targeted suicide prevention and mental health interventions.

### Contributions

Table 1 summarizes the main contributions and novelty of the study across 6 key dimensions: data source, demographics, perpetrator relationships, abuse types, methodology, and implications. For each dimension, we describe the specific contribution of this work and the corresponding gap in the existing literature that it addresses.

**Table 1:** Summary of study contributions and gaps addressed.

Aspect	Contribution	Novelty or gap addressed
Data source	Analyzed 27,434 Reddit posts; identified 975 posts explicitly mentioning family abuse in suicidal ideation	No prior studies have specifically examined family abuse in suicidal posts on social media.
Demographics	Extracted self-reported age and gender; highlighted high representation of young adults and LGBTQ+^a^ individuals	Prior work focused on detecting suicidal content rather than examining demographic patterns in abuse-related posts.
Perpetrator relationships	Identified abuse across parents, siblings, partners, stepparents, and extended family	First systematic analysis of relational contexts in social media posts reporting suicidal ideation.
Abuse types	Characterized physical, emotional, and sexual abuse and neglect with associated keywords and frequency counts	No prior social media studies mapped abuse types in posts reporting suicidal ideation.
Methodology	Developed an LLM^b^-supported keyword- and context-based extraction module to detect abuse mentions and perpetrators	Novel approach for systematic extraction from social media text, combining LLM capabilities with rule-based methods.
Implications	Insights derived from this study may help guide the development of interventions for groups at higher risk of familial abuse and suicidal ideation	Demonstrates how insights from social media posts can inform intervention strategies for familial abuse–related suicidal ideation, an approach not previously explored.

^a^LGBTQ+: lesbian, gay, bisexual, transgender, queer or questioning, and other sexual and gender identities.

^b^LLM: large language model.

## Methods

### Study Overview

Figure 1 provides an overview of the study workflow. Starting from the Reddit *SuicideWatch* corpus, we first identified family abuse–related suicidal ideation posts using a keyword-based selection process informed by manual review, large language model (LLM)–assisted keyword expansion, and human validation. The identified corpus was then analyzed across 3 dimensions: demographic characteristics, forms of family abuse, and perpetrator categories.

**Figure 1:**
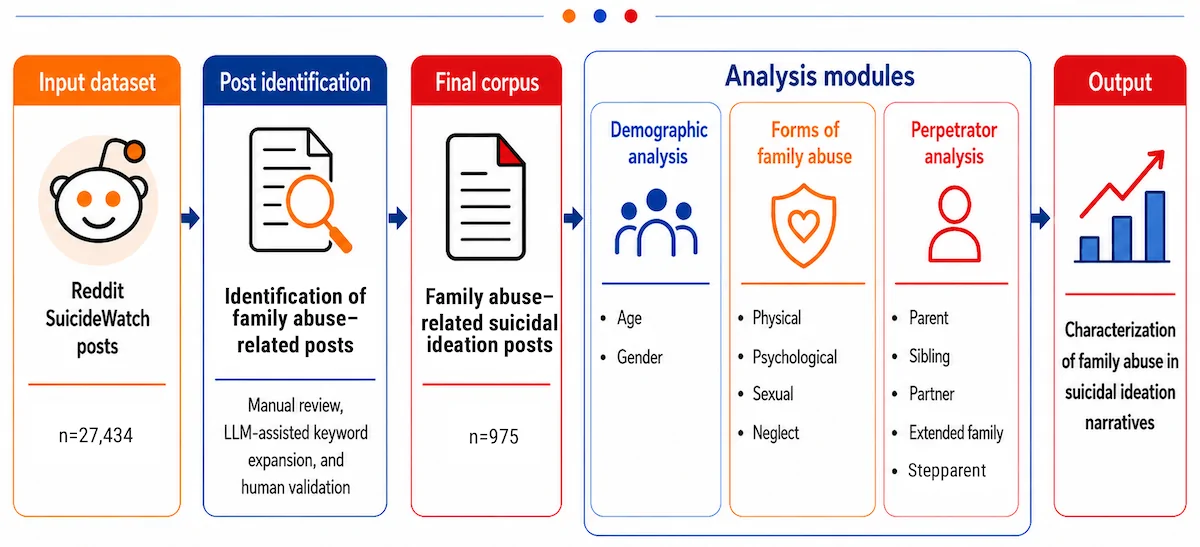
Overall workflow for identifying and characterizing family abuse–related suicidal ideation posts. LLM: large language model.

### Dataset Creation and Description

#### Collection of Suicidal Ideation Posts

The suicidal ideation posts analyzed in this study were sourced from Reddit, a widely used social media platform where users engage in discussions on diverse topics. The corpus used in this study was developed in our previous work [27] using a publicly available Reddit dataset hosted on Kaggle. The original dataset contained approximately 230,000 Reddit posts, including suicidal posts collected from the *r/SuicideWatch* subreddit and nonsuicidal posts collected from multiple other subreddits. As the present study focuses on suicidal discourse, only posts from *r/SuicideWatch* were included, resulting in a corpus of 27,434 posts.

The *r/SuicideWatch* subreddit is a discussion forum where users anonymously share experiences related to suicidal ideation and severe psychological distress. Posts in this forum typically consist of personal experiences, thoughts, and help-seeking behaviors, providing largely unfiltered accounts of distress and family abuse as reported directly by affected individuals. As a result, posts from *r/SuicideWatch* provide a rich source of naturally occurring text that facilitates the analysis of family abuse in the context of suicidal ideation.

The posts were published between December 16, 2008, and January 2, 2021, covering a broad temporal span. The dataset consists solely of original user posts, excluding all responses and comments. During preprocessing, only English-language posts containing meaningful textual content were retained.

#### Selection of Suicidal Posts Involving Family Abuse

Manually reviewing these 27,434 suicidal posts to identify incidents related to family abuse would be highly resource intensive, particularly given that each post contains, on average, more than 700 words. To address this challenge, an automated keyword-based selection approach was used, where initial keywords are derived through manual review and later extended by an LLM to ensure broad coverage of expressions related to family abuse. Although keyword-based filtering has limitations in contextual understanding, the substantial reduction in manual effort, especially in the initial screening phase, makes it a feasible and widely adopted method for large-scale data selection and annotation [28,29].

#### Keyword Selection Procedure

##### Overview

The keyword selection process follows a systematic procedure designed to ensure both precision and comprehensive coverage. The procedure consists of 2 phases. In phase 1, a manual review is performed on a subset of posts to identify naturally occurring terms and expressions associated with family abuse. In phase 2, an LLM is used to generate additional relevant keywords, expanding coverage to include variations and contextually related expressions not captured in the initial manual review.

##### Phase 1: Manual Review

A representative subset of 2000 posts, randomly selected from the corpus, was manually reviewed to ensure an unbiased sample. Each post in the corpus was assigned a unique identifier, and 2000 identifiers were selected using a random number generator, the *random.sample()* function of Python (Python Software Foundation), so that every post had an equal chance of inclusion. This subset was chosen to enable detailed examination of naturally occurring expressions of family abuse across diverse contexts in the corpus.

The manual review and keyword extraction in phase 1 were conducted by 2 trained annotators with expertise in social media discourse and mental health: annotator 1, who has more than 8 years of experience analyzing social media and mental health–related content, and annotator 2, who has several years of experience analyzing social media data.

During phase 1, reviewers extracted phrases that explicitly or implicitly referred to abusive behaviors, harmful family dynamics, or distressing household environments and compiled them into an initial candidate keyword set. This phase was designed as an exploratory keyword-generation step rather than a fixed-label double-coding task.

The goal was to identify candidate family abuse–related expressions, not to assign abuse or nonabuse labels or abuse-type labels to a fixed set of posts. Therefore, Cohen κ was not calculated for phase 1 because there was no fixed set of categorical labels or shared annotation units on which agreement could be computed. To ensure consistency, reviewers followed predefined inclusion criteria and retained only terms whose relevance to family abuse was clear and unambiguous, as described below. Any discrepancies were resolved through discussion until consensus was reached.

Keywords for types of abuse included terms representing different forms of abuse, including emotional, physical, and financial abuse and neglect. Keywords for relational contexts included terms indicating specific familial roles and dynamics, such as *abusive parent*, *toxic household*, or *manipulative sibling*, highlighting interactions between perpetrators and individuals experiencing abuse. Keywords for experience of abuse included phrases reflecting the emotional and psychological toll of abuse, such as *helpless at home*, *scared to go home*, and *living in fear.* Other keywords included terms not covered by the above categories but still relevant to family abuse.

##### Phase 2: LLM-Based Keyword Expansion

The initial keyword set from the manual review was subsequently expanded in phase 2 using an LLM to capture additional terms and contextual variations. We used ChatGPT (GPT-4o; OpenAI), an LLM, to generate additional keywords relevant to family abuse; the following prompt was used to produce a set of keywords: “Provide a list of comma-separated keywords associated with family abuse including terms related to domestic violence, abusive family structures, and various forms of abuse such as emotional, physical, sexual, and verbal abuse. Focus on terms like abusive parents, sibling abuse, gaslighting, psychological manipulation, family trauma, and the impacts of living in fear or experiencing an unsafe home environment.”

The LLM-generated keywords (not already included in the phase 1 manual set) were validated for contextual relevance by the senior annotator (annotator 1). For each keyword, the first 10 occurrences in the dataset (or all occurrences if fewer than 10) were examined. This sample size was chosen to balance efficiency and representativeness, ensuring that retained keywords accurately reflected typical contexts. A keyword was retained only if at least 70% of its sampled occurrences explicitly referenced family abuse, ensuring high contextual relevance and reliability. The validated keywords were then combined with the initial set from phase 1 to form the final comprehensive, nonredundant keyword list. Note that the LLM was not used to directly classify posts as family abuse–related or nonfamily abuse–related. Instead, it was used only to expand the manually derived keyword list by providing additional candidate terms.

The phase 1 manual review produced 53 initial candidate keywords and phrases. In phase 2, the LLM generated 114 candidate keywords and phrases, of which 26 nonredundant terms were retained after duplicate or overlapping terms were removed and contextual relevance was validated using the 70% threshold. After combining the phase 1 terms with the retained phase 2 terms, the final postselection keyword list contained 79 nonredundant keywords and phrases (presented in Multimedia Appendix 1).

#### Description of the Final Dataset

Applying the final comprehensive keyword list derived from the keyword selection procedure (refer to the Phase 1 and Phase 2 subsections) to the full corpus, 975 posts were identified explicitly referencing family abuse. The resulting corpus has a mean word count of 730.27 (SD 609.75) per post. It is important to note that while this keyword-based approach captures a broad range of family abuse–related expressions in suicidal posts, it may not identify posts that use indirect, unconventional, or rare language to describe abusive experiences. The top family abuse–related keywords are shown in Figure 2.

**Figure 2:**
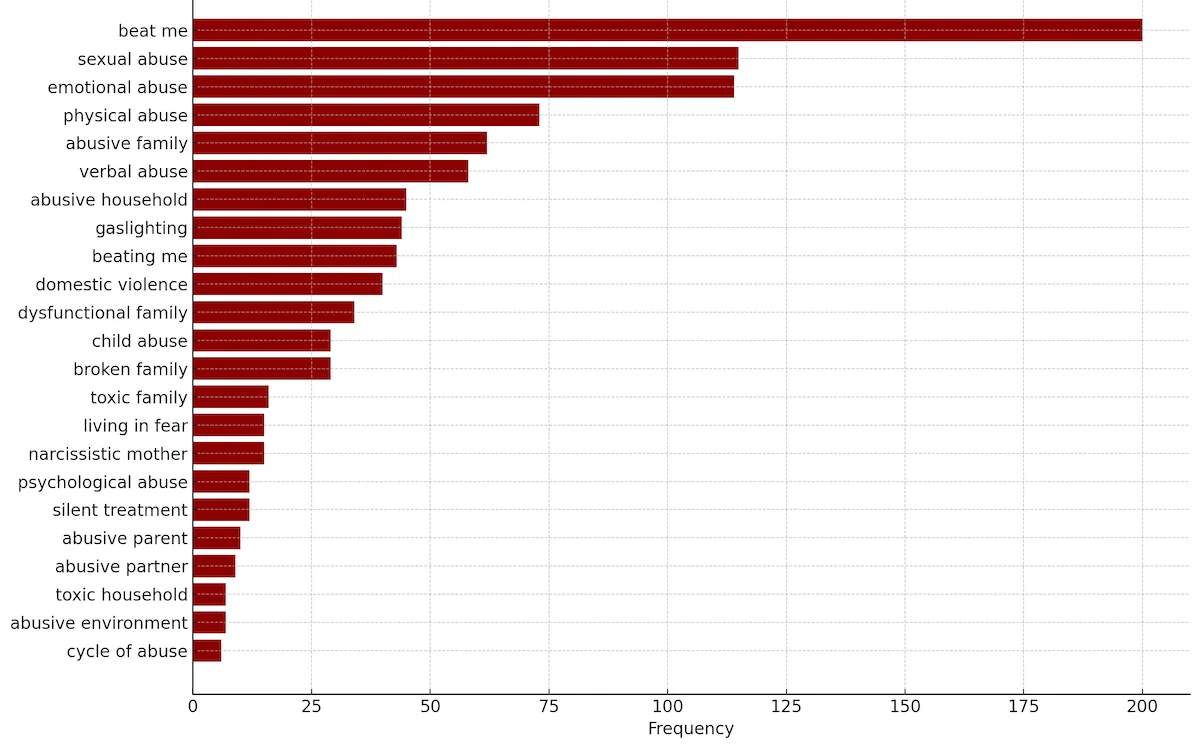
Distribution of family abuse–related keywords in the corpus. Frequency indicates the number of posts in which each keyword appears.

### Demographic Characteristics of Individuals Reporting Family Abuse

#### Overview

We examined the demographic characteristics, specifically gender and age, of individuals reporting suicidal ideation in the context of family abuse. To extract age and gender information, a self-reported demographic information extraction tool, SocialDemoExtract, developed by our research group [27], was used. The tool uses a combination of keywords and rule-based patterns to identify demographic information.

#### Age Distributions

The age distribution of individuals who experienced family abuse was analyzed to understand their representation across age groups. Age groups were categorized as adolescence (aged 13-17 years), young adulthood (aged 18-24 years), early to middle adulthood (aged 25-39 years), and middle-aged adulthood (aged 40-59 years), consistent with the approach used by Wang et al [30]. The other category includes individuals aged <13 years and those aged ≥60 years.

#### Gender Distributions

Family abuse affects individuals across all genders, though its prevalence and forms vary [31]. To examine gender-related patterns in this dataset, self-reported gender and identity mentions were identified and categorized into three groups: (1) men, (2) women, and (3) people who identify as lesbian, gay, bisexual, transgender, queer or questioning, and other sexual and gender identities (LGBTQ+).

### Forms of Family Abuse

#### Overview

Familial abuse encompasses a range of harmful behaviors, including neglect, emotional manipulation, sexual abuse, and physical violence, all of which have been strongly associated with suicidal ideation in a population-based study by Calder et al [32]. A deeper understanding of the frequency, context, and nuances of abuse, explored through social media discourse analysis, can offer valuable insights into these distributions, thereby complementing clinical and population-based studies and informing the development of targeted, evidence-based interventions. To support this analysis, keywords for each form of abuse were curated using ChatGPT (GPT-4; OpenAI), generating comprehensive lists of formal terms, social media expressions, slang, and abbreviations. All candidate keywords for the different abuse types were manually validated for contextual relevance before inclusion. For each keyword, the first 10 occurrences in the dataset were examined, and a keyword was retained only if at least 70% of these occurrences clearly represented the intended form of abuse, excluding metaphorical, ambiguous, or unrelated uses. After validation, the final category-specific lists contained 47 physical abuse terms, 53 emotional or psychological abuse terms, 45 sexual abuse terms, and 40 neglect terms; the full lists are presented in Multimedia Appendix 1. The validated category-specific keyword lists were then used to assign abuse-type categories to posts based on the presence of keywords from the corresponding abuse category. Therefore, the resulting abuse-type assignments should be interpreted as scalable keyword-supported categorizations based on manually validated keyword lists, rather than as independently assigned manual post-level labels. This approach was used to enable systematic analysis of a larger corpus while retaining human validation at the keyword selection stage.

#### Physical Abuse

Physical abuse involves the deliberate use of physical force to inflict harm or injury upon a family member. This category includes actions such as hitting, slapping, punching, beating, and choking, which are frequently used as means of control or intimidation. Some example keywords include *physical abuse*, *hitting*, *slapping*, *punching*, *beating*, *choking*, *bruises*, and *physical assault* (the full list is presented in Multimedia Appendix 1).

#### Emotional or Psychological Abuse

Emotional or psychological abuse refers to behaviors that undermine an individual’s emotional well-being and sense of self-worth. These behaviors include verbal assaults, gaslighting, threats, humiliation, and manipulation, all of which are intended to instill fear, shame, or insecurity in the individual experiencing abuse. Some associated keywords for emotional abuse considered in this study include *emotional abuse*, *psychological abuse*, *verbal assault*, *gaslighting*, *threats*, *humiliation*, *manipulation*, and *belittling* (the full list is presented in Multimedia Appendix 1).

#### Sexual Abuse

Sexual abuse within the family refers to nonconsensual sexual behaviors or exploitation, including incest, molestation, inappropriate touching, sexual assault, and forced sexual activity. These acts occur without the individual’s consent and often involve coercion, manipulation, or the exploitation of trust [33]. Some keywords used to identify such sexual abuse include *sexual abuse*, *molestation*, *incest*, *inappropriate touching*, *sexual assault*, *sexual exploitation*, and *forced sex* (the full list is presented in Multimedia Appendix 1).

#### Neglect

Neglect occurs when a caregiver fails to provide essential care or resources—such as food, medical attention, emotional support, or shelter—to a dependent family member. This form of abuse can result in long-term physical, emotional, and psychological consequences. Some keywords used to identify neglect include *neglect*, *abandonment*, *lack of food*, *parent neglect*, and *child neglect* (the full list is presented in Multimedia Appendix 1).

### Perpetrators of Family Abuse

#### Overview

We aim to identify which family members—parents, siblings, or intimate partners—are most frequently associated with abuse among individuals experiencing suicidal ideation, with a focus on understanding the relational dynamics involved.

#### Parents

Parents are commonly identified as perpetrators of abuse within the family context. Research shows that parental abuse often stems from stressors such as financial hardship, mental health challenges, or substance use and can lead to long-term consequences for children, including increased susceptibility to suicidal ideation [34]. Parental neglect, which often co-occurs with abuse, is also associated with developmental delays and elevated mental health risks. The following keywords were used to identify parental figures: *mom*, *dad*, *mother*, *father*, *parents*, *mum*, *mama*, *papa*, *ma*, *pa*, *dada*, and *mummy*.

#### Intimate Partners

Intimate partner violence (IPV) is a well-established form of abuse that disproportionately affects women, although men can also experience IPV [35,36]. IPV is strongly associated with suicidal ideation, particularly when emotional and physical abuse are persistent and severe [37]. The following terms were used to identify intimate partners: *partner*, *boyfriend*, *girlfriend*, *husband*, *wife*, *spouse*, *significant other*, *lover*, *companion*, *fiancée*, *fiancé*, *gf*, and *bf*.

#### Siblings

Sibling abuse, though less frequently studied, can significantly contribute to family trauma. It often involves emotional manipulation, bullying, or physical violence and is strongly linked to depressive symptoms and suicidal ideation [38]. The following terms were used to identify siblings: *brother*, *sister*, *sibling*, *bro*, *sis*, *half-brother*, *half-sister*, *stepbrother*, and *stepsister*.

#### Stepparents

Stepparents often play complex roles in family dynamics, and their involvement in abuse is linked to strained relationships with stepchildren [39,40]. Such abuse may include neglect, emotional abuse, or physical violence, particularly when children feel alienated or rejected. The following terms were used to identify stepparents: *stepfather*, *stepmother*, *stepdad*, and *stepmom*.

#### Extended Family Members

Extended family members, including grandparents, uncles, aunts, and cousins, can also be involved in family abuse. The following terms were used to identify such perpetrators: *grandfather*, *grandmother*, *grandpa*, *grandma*, *uncle*, *aunt*, *cousin*, *nephew*, *niece*, *in-law*, *brother-in-law*, *sister-in-law*, *mother-in-law*, and *father-in-law*.

#### Perpetrator Identification and Validation

To identify perpetrators of family abuse, a keyword- and rule-based module was developed to extract potential perpetrator mentions from posts. The module first detects familial terms (eg, *father*, *mother*, *parent*, *sibling*, and *stepparent*) and then searches for family abuse keywords—using the same list applied to identify family abuse–related posts—within a window of ±1 sentence, with sentence boundaries determined by spaCy’s (Explosion) sentence splitter. The identified familial term–abuse keyword pairs were flagged as candidate perpetrator mentions and subsequently verified by a senior annotator (annotator 1) to ensure that the familial term explicitly referred to the perpetrator.

Verified candidate perpetrator mentions were then aggregated at the post level so that each perpetrator category was counted at most once per post.

An additional sample-based interannotator agreement analysis was conducted to assess the reliability of the perpetrator verification procedure. A second annotator independently reviewed 617 candidate perpetrator mentions identified by the keyword- and rule-based module from a random sample of 195 posts, representing 20% of the family abuse–related corpus. Each candidate was coded as valid or not valid based on whether the familial term explicitly referred to the perpetrator in context. The annotators agreed on 572 of 617 candidates, including 205 valid and 367 nonvalid cases, yielding 92.71% agreement and a Cohen κ of 0.84. This reliability check indicated high agreement and was used to assess the consistency of the verification procedure; the second annotator’s labels were not used to reassign perpetrator categories or recalculate the prevalence estimates reported in the Results section.

### Ethical Considerations

No human participants were recruited or directly involved in this study. The study analyzes only publicly available, anonymized Reddit posts related to suicidal ideation, and the original corpus does not contain personally identifiable information. No direct quotes containing identifiable details are presented.

## Results

### Demographic Characteristics of Individuals Reporting Family Abuse

Table 2 presents the self-reported gender and age distribution of suicidal individuals who reported experiences of family abuse.

**Table 2:** Self-reported demographic characteristics of family abuse–related suicidal ideation posts.

Characteristic		Posts, n (%)^a^
**Gender and/or identity (n=150)**		
	Men	86 (57.3)
	Women	47 (31.3)
	LGBTQ+	17 (11.3)
**Age (n=313)**		
	Adolescents (13-17 years)	78 (24.9)
	Young adults (18-24 years)	144 (46.0)
	Early to mid-adulthood (25-39 years)	78 (24.9)
	Middle-aged adults (40-59 years)	6 (1.9)
	Other (<13 or ≥60 years)	7 (2.2)

^a^Percentages are calculated within posts containing self-reported information for the corresponding demographic characteristic.

SocialDemoExtract identified self-reported gender information in 150 posts. Among these, 57.3% (n=86) were authored by individuals identifying as men, 31.3% (n=47) by those identifying as women, and 11.3% (n=17) by individuals identifying as LGBTQ+.

SocialDemoExtract shows that, of the 975 posts, 313 contain self-reported age information. Among the 313 posts with self-reported age information, young adults (aged 18-24 years) accounted for the largest proportion, with 144 (46.0%) posts, followed by adolescents (aged 13-17 years) with 78 (24.9%) posts. In contrast, middle-aged adults (aged 40-59 years) and individuals in the other age category (aged <13 or ≥60 years) are underrepresented.

### Forms of Family Abuse

Table 3 shows the distribution of familial abuse types identified in suicidal posts, emphasizing their prevalence. Each abuse category is represented by post counts and percentages, while associated keywords are reported using occurrence counts, offering insights into the nature, frequency, and severity of these self-reported experiences.

Physical abuse (683 posts) emerged as the most frequently reported category, with high prevalence of terms such as beat me (146 occurrences) and physical abuse (80 occurrences). Specific expressions such as beating (54 occurrences), hit me (53 occurrences), and kicked (40 occurrences) reflect common violent behaviors.

Emotional or psychological abuse (350 posts) also appeared prominently, with terms such as emotional abuse (119 occurrences) and verbal abuse (62 occurrences) being frequently mentioned. Other expressions, including gaslighting (36 occurrences), yelling (36 occurrences), and insults (15 occurrences), further underscore the psychological harm.

Sexual abuse (296 posts) is primarily represented by the terms sexual abuse (125 occurrences) and rape (93 occurrences), along with molested (25 occurrences) and sexual assault (20 occurrences).

Neglect (126 posts) is reflected in terms such as neglect (63 occurrences), abandoned (35 occurrences), and abandonment (17 occurrences).

Table 4 provides examples of text excerpts from posts illustrating various types of family abuse.

**Table 3:** Distribution of family abuse types identified in suicidal ideation posts (N=975).^a^

Abuse type	Posts, n (%)
Physical abuse	683 (70.1)
Emotional or psychological abuse	350 (35.9)
Sexual abuse	296 (30.4)
Neglect	126 (12.9)

**^a^**Abuse type categories were not mutually exclusive; a post could be assigned to more than one category.

**Table 4:** Example excerpts illustrating the forms of family abuse identified in suicidal ideation posts.

Type of abuse	Example
Physical	“...When I was a kid my mom used to abuse me relentlessly. She would beat me with belts and extension cords, and punch me in the face and tell me I’m stupid, and burned me with cigarettes a few times...”
Emotional or psychological	“...Anyways, I have an emotionally abusive dad, who constantly calls my brothers and I jackasses, shithead, dumbasses, and he calls me a bitch all the time…”
Sexual	“...I was sexually abused when I was 4-6 years old by my older male cousin...”
Neglect	“...alcoholic enabling father gave me a childhood that was so filled with neglect...”

### Perpetrators of Family Abuse

Table 5 presents the post-level distribution of perpetrator categories and associated keywords. A perpetrator category was counted once per post if at least 1 familial term from that category was identified in an abuse-related context and verified as referring to the perpetrator. Therefore, the counts reflect the number of posts mentioning each perpetrator category, not the total number of keyword occurrences.

Parent (435 posts; 435/975, 44.6%; 95% CI 41.52%-47.75%) was a predominant theme, with suicidal individuals most frequently mentioning *mom* (112 occurrences), *mother* (86 occurrences), and *dad* (84 occurrences), followed by *parents* (67 occurrences), *father* (56 occurrences), *parent* (22 occurrences), and *mum* (9 occurrences), reflecting diverse references to parental figures in abusive contexts.

Mentions of sibling abuse (104 posts; 104/975, 10.7%; 95% CI 8.88%-12.76%) most frequently involved *brother* (50 occurrences) and *sister* (32 occurrences), with occasional references to *siblings*, *stepbrother*, or other specific cases.

Partner (37 posts; 37/975, 3.8%; 95% CI 2.77%-5.19%) abuse in intimate partnerships was reflected by mentions of *girlfriend* (13 occurrences), *husband* (11 occurrences), and *wife* (8 occurrences), with less frequent informal references such as *gf* (4 occurrences) and *bf* (1 occurrence).

Abuse by extended family members (34 posts; 34/975, 3.5%; 95% CI 2.51%-4.83%) was most frequently associated with *uncle* (12 occurrences) and *grandparents* (7 occurrences), with occasional mentions of *aunt* (5 occurrences), *cousin* (4 occurrences), *grandpa* (3 occurrences), and *grandma* (3 occurrences).

Stepparent abuse (12 posts; 12/975, 1.23%; 95% CI 0.71%-2.14%) was less frequently mentioned, most often involving *stepdad* (8 occurrences), followed by *stepmother* (3 occurrences) and *stepfather* (1 occurrence).

Table 6 presents example excerpts illustrating the perpetrator categories identified in family abuse–related suicidal ideation posts.

**Table 5:** Perpetrator categories identified in family abuse–related suicidal ideation posts (N=975).^a^

Perpetrator category	Posts, n (%)	Most frequent associated terms, n
Parent	435 (44.6)	mom: n=112 mother: n=86 dad: n=84 parents: n=67 father: n=56 parent: n=22 mum: n=9
Sibling	104 (10.7)	brother: n=50 sister: n=32 sibling: n=14 siblings: n=5 stepbrother: n=1
Partner	37 (3.8)	girlfriend: n=13 husband: n=11 wife: n=8 gf: n=4 bf: n=1
Extended family	34 (3.5)	uncle: n=12 grandparents: n=7 aunt: n=5 cousin: n=4 grandpa: n=3 grandma: n=3
Stepparent	12 (1.2)	stepdad: n=8 stepmother: n=3 stepfather: n=1

^a^Perpetrator categories were counted at the post level, with each category counted at most once per post. Categories were not mutually exclusive; therefore, a post could mention more than one perpetrator category.

**Table 6:** Example excerpts illustrating perpetrator categories in family abuse–related suicidal ideation posts.

Perpetrator type	Example
Parent	“...My father was an alcoholic as well as *abusive*, after he died when I was 12...”
Sibling	“...My *older brother* also used to *beat me up* when I was little...”
Partner	“...My *boyfriend* started to *control me by gaslighting*, stonewalling, denying my needs, withholding sex and affection...”
Extended family	“...My uncle violently *raped* me from the ages of 7 to 13 while my aunt watched and/or held me down...”
Stepparent	“...My *stepdad* started physically and mentally *abusing* me starting around ages 5 or 6. the physical abuse slowed way down when i hit puberty and he moved to *touching me* instead of hitting me...”

## Discussion

### Principal Results

Among posts with self-reported age information, young adults and adolescents were the most frequently represented age groups. This suggests that online suicidal ideation posts may offer useful descriptive insight into abuse-related distress among younger users, who may be more inclined to disclose personal experiences in online spaces.

The lower representation of older adults may reflect reduced social media engagement among older populations [41] or a lower likelihood of disclosing sensitive family abuse experiences in online settings. Because demographic information was available only when users self-disclosed it, these findings should be interpreted as patterns in disclosed online narratives rather than as population-level prevalence estimates.

Among posts with available gender and identity information, users identifying as men represented the largest group, followed by users identifying as women and those with LGBTQ+ self-disclosures. This pattern may reflect disclosure behaviors specific to anonymous online communities. The presence of LGBTQ+ self-disclosures suggests that online forums may capture abuse-related suicidal narratives from groups that are particularly vulnerable to family rejection, stigma, or interpersonal stress, although this finding should be interpreted cautiously given that demographic disclosure was incomplete.

Physical abuse was the most frequently identified abuse type, followed by emotional or psychological abuse, sexual abuse, and neglect. This pattern is consistent with prior research linking physical, sexual, and emotional or psychological abuse to suicidal ideation and suicidal behaviors [9,42]. These findings suggest that family abuse–related suicidal ideation posts frequently reference more than 1 form of abuse, indicating the heterogeneous nature of family abuse described in online mental health narratives.

Parental relationships were the most frequently identified perpetrator category, followed by siblings, intimate partners, extended family members, and stepparents. The predominance of parental perpetrators is consistent with prior research highlighting the central role of parent-child dynamics in family abuse and its association with suicidal ideation [6,11]. The presence of multiple perpetrator categories further indicates that abuse-related suicidal narratives are not restricted to a single family role or relationship type but instead reflect the varied and complex household dynamics in which individuals describe abuse-related distress.

### Translational Implications

This study has practical implications for computational mental health research and online support systems. The findings show that suicidal ideation posts involving family abuse often contain identifiable abuse-related terms and references to family relationships.

These features may help researchers organize large collections of online mental health posts into interpretable themes, such as physical abuse, emotional or psychological abuse, sexual abuse, neglect, and perpetrator relationship categories.

The findings may also inform the design of online support resources. For example, posts describing unsafe home environments, parental abuse, or ongoing family violence may require support resources that extend beyond general suicide prevention messaging to include information about family violence, abuse support services, and crisis resources. In this way, aggregate analysis of online narratives can help identify the types of support needs that users disclose in public mental health forums.

These implications should be interpreted cautiously. Because this study provides descriptive, aggregate-level analysis of public online disclosures, the findings should not be interpreted as clinical assessment, diagnosis, or individual-level suicide risk prediction.

### Limitations

This study has several limitations that should be considered when interpreting the findings. The analysis relies on self-reported English-language posts from the Reddit *SuicideWatch* forum, where users may omit details, describe experiences selectively, or frame events differently than they would in clinical or survey settings. Social media disclosures may also reflect platform-specific norms, limiting generalizability to other languages, cultural contexts, online communities, or offline populations. In addition, demographic information such as age and gender was available only when users explicitly disclosed it, limiting subgroup-level comparisons and the interpretation of demographic patterns. Finally, this study is descriptive in nature and does not establish a causal relationship between family abuse and suicidal ideation.

### Conclusions

This study analyzed 975 suicidal ideation posts involving family abuse to characterize demographic patterns, forms of abuse, and relationships to perpetrators in online mental health narratives. The findings show that family abuse–related suicidal discourse contains diverse abuse-type and relational-context signals, with physical abuse and parental relationships appearing most frequently.

Overall, this work demonstrates the potential of computational social media analysis to support descriptive, hypothesis-generating research on family abuse and suicidal ideation. Future research may examine how family abuse co-occurs with other psychosocial stressors and how these patterns evolve over time in online suicidal ideation narratives.

## Appendix

Multimedia Appendix 1 Full keyword lists and supplementary materials, including (1) the complete 79-term postselection keyword list used to identify family abuse–related suicidal ideation posts; (2) the large language model prompts used to generate candidate keywords for each abuse type; (3) the category-specific keyword lists for physical abuse (47 terms), emotional or psychological abuse (53 terms), sexual abuse (45 terms), and neglect (40 terms); and (4) examples of age and gender information extracted using SocialDemoExtract.

## Abbreviations

ACE: adverse childhood experience
IPV: intimate partner violence
LGBTQ+: lesbian, gay, bisexual, transgender, queer or questioning, and other sexual and gender identities
LLM: large language model
RQ: research question

## Data Availability

The raw Reddit posts used in this study were obtained from a publicly available Reddit dataset. The keyword lists used for post selection and abuse-type categorization are provided in Multimedia Appendix 1. Aggregate counts, nonidentifying summary outputs, and analysis code will be made available through the project GitHub repository upon publication. Additional derived materials are available from the corresponding author upon reasonable request.
