# Supplementary material for: Characterizing Family Abuse in Suicidal Ideation Posts on Reddit: Large Language Model–Assisted Content Analysis
Source: JMIR Infodemiology. 2026 Sep 14;6:e90340. doi: 10.2196/90340 (PMC13620435; doi:10.2196/90340)
Supplement: Supplementary file 1 [file infodemiology_v6i1e90340_app1.docx]

**Full Keyword Lists for Identifying Suicidal Ideation Posts Related to Family Abuse**

**Keywords**: family abuse, domestic violence, abusive family, abusive household, toxic household, toxic family, parental abuse, sibling abuse, child abuse, spousal abuse, elder abuse, emotional abuse, physical abuse, sexual abuse, verbal abuse, psychological abuse, financial abuse, coercive control, abusive step-parent, abusive partner, abusive spouse, violent parent, violent sibling, controlling family, manipulative family, manipulative sibling, narcissistic parent, narcissistic mother, narcissistic dad, narcissistic mom, overbearing parent, abusive upbringing, family exploitation, living in fear, scared to go home, unsafe at home, violence at home, beaten by family, hurt by family, bruises from family, scars from family, helpless at home, pain at home, can’t leave home, stuck with them, shattered family, broken family, surviving abuse, abuse survivor, victim of family abuse, traumatized by family, humiliated at home, shamed by family, disowned by family, silent treatment, locked at home, oppression at home, family intimidation, ignored cries for help, threats at home, yelling and hitting, constant yelling, family fights, domestic disputes, family neglect, cruel family, harmful family, dysfunctional family, family arguments, family bullying, family threats, intergenerational trauma, cycle of abuse, family trauma, family violence, hidden abuse, not the best household, beat me, beating me.

**LLM Prompts Designed for Various Forms of Abuse**

Prompt for physical abuse:

Generate a comprehensive list of keywords, phrases, and expressions that indicate physical abuse. Include formal terms (eg, physical abuse, assault), descriptive phrases people might use on social media (eg, “hit me,” “choked”), slang, abbreviations, and variations. Exclude emotional abuse, sexual abuse, and neglect terms. Provide the output as a Python list, with each term in quotes.

Prompt for emotional/psychological abuse:

Generate a comprehensive list of keywords, phrases, and expressions that indicate emotional or psychological abuse. Include formal terms (eg, emotional abuse, psychological abuse), common social-media expressions, slang, abbreviations (eg, “psych abuse”), and subtle manipulative behaviors. Exclude physical abuse, sexual abuse, and neglect terms. Provide the output as a Python list, with each term in quotes.

Prompt for sexual abuse:

Generate a comprehensive list of keywords, phrases, and expressions that indicate sexual abuse or assault. Include formal terms (eg, sexual abuse, sexual assault, child sexual abuse), social-media phrasing (eg, “touched me inappropriately,” “coerced sex”), slang, abbreviations (eg, CSA, SA), and child/adult contexts. Exclude physical abuse, emotional abuse, and neglect terms. Provide the output as a Python list, with each term in quotes.

Prompt for neglect:

Generate a comprehensive list of keywords, phrases, and expressions that indicate neglect. Include formal terms (eg, neglect, child neglect, medical neglect), social-media phrasing (eg, “left alone for hours,” “no food at home”), slang, and descriptive phrases related to lack of supervision, food, shelter, clothing, and medical care. Exclude physical abuse, sexual abuse, and emotional abuse terms. Provide the output as a Python list, with each term in quotes.

**Keyword Lists for Identifying Various Types of Family Abuse**

To systematically identify mentions of family abuse in online posts, we curated extensive keyword lists for four abuse categories.

Keywords for physical abuse: physical abuse, physically abused, hurt me, hit me, hitting, slapped, slapping, punching, punched, beating, beat me, choking, choked, kicking, kicked, dragged, thrown around, bruises, bruised, marks on my body, injuries, broken bones, fractures, physical harm, violent behavior, physical punishment, assaulted, assault, smacked, rough handling, manhandled, shoved, pushed me, violent outbursts, domestic violence, dv, physically hurt, physically attacked, grabbed me hard, hair pulling, pulled my hair, forced restraint, held me down, hit with objects, belt beating, whipped, spanked hard.

Keywords for emotional/psychological abuse: emotional abuse, psychological abuse, psych abuse, verbal abuse, verbal assault, gaslighting, threats, humiliation, belittling, manipulation, manipulative behavior, constant criticism, criticizing me, degrading language, insults, name calling, fear tactics, mind games, emotional blackmail, silent treatment, stonewalling, invalidating my feelings, controlling behavior, extreme jealousy, monitoring my messages, blaming me for everything, shaming, guilt-tripping, mocking, yelling, screaming at me, demeaning, toxic behavior, mental torture, mental cruelty, put me down, embarrassing me, public humiliation, calling me worthless, criticizing everything I do, intimidation, threatening me, manipulative tactics, emotional manipulation, psychological torment, invalidating me, over-controlling, obsessive criticism, passive-aggressive behavior, withholding affection, managing emotions, constant monitoring, emotional coercion.

Keywords for sexual abuse: sexual abuse, sexually abused, molestation, molested, incest, inappropriate touching, touched me inappropriately, sexual assault, assaulted sexually, coerced sexual behavior, coerced sex, pressured into sex, forced sexual activity, forced sex, rape, raped, grooming, sexual exploitation, child sexual abuse, csa, sexual harassment, harassed sexually, unwanted advances, non-consensual touching, nonconsensual sex, sexual coercion, marital rape, fondling, touching without consent, forced kissing, forced nudity, exposure, flashing, stealthing, drugged and assaulted, spiked drink assault, tricked into sexual acts, abusive sexual behavior, sexual victimization, unwanted sexual contact, sexually victimized, child molestation, sexual intimidation, sexualized manipulation, sexual grooming.

Keywords for neglect: neglect, neglected, child neglect, parental neglect, abandonment, abandoned, lack of food, no food at home, starved, malnourished, lack of shelter, homeless because of parents, unsafe living conditions, dangerous home, lack of clothing, insufficient clothing, inadequate clothing, medical neglect, no medical care, ignored injuries, failure to provide medical attention, emotional neglect, ignored emotionally, nobody cares about me, absence of affection, failure to provide basic needs, not taken care of, left alone for hours, no supervision, failure to supervise, left to raise myself, parents never home, missed school because of parents, ignored cries for help, absence of caregiving, lack of guidance, unsafe environment, neglected health, ignored developmental needs, inadequate supervision.

**Examples of Extracted Age and Gender Information**

Table A1. Examples of extracted age and gender information using SocialDemoExtract

| **Text** | **Extracted Age and Gender** |
| --- | --- |
| I’m a 15-year-old girl that has excessive body hair. | 15, Female |
| I’m 21 on June 24th. | 21, _ |
| I’m 19. | 19, _ |
| I’m a 22-year-old male in a long-distance relationship with my girlfriend of 6 months, 20 years old. | 22, Male |
| I’m about to turn 27. | 27, _ |
| I’m only 19. | 19, _ |
| I am a 23-year-old male. | 23, Male |
